# Double Flash Illusions: Current Findings and Future Directions

**DOI:** 10.3389/fnins.2020.00298

**Published:** 2020-04-03

**Authors:** Julian Keil

**Affiliations:** Biological Psychology, Christian-Albrechts-Universität zu Kiel, Kiel, Germany

**Keywords:** double flash illusion, multisensory integration, crossmodal influence, perception, congruence, sound-induced flash illusion

## Abstract

Twenty years ago, the first report on the sound-induced double flash illusion, a visual illusion induced by sound, was published. In this paradigm, participants are presented with different numbers of auditory and visual stimuli. In case of an incongruent number of auditory and visual stimuli, the influence of auditory information on visual perception can lead to the perception of the illusion. Thus, combining two auditory stimuli with one visual stimulus can induce the perception of two visual stimuli, the so-called fission illusion. Alternatively, combining one auditory stimulus with two visual stimuli can induce the perception of one visual stimulus, the so-called fusion illusion. Overall, current research shows that the illusion is a reliable indicator of multisensory integration. It has also been replicated using different stimulus combinations, such as visual and tactile stimuli. Importantly, the robustness of the illusion allows the widespread use for assessing multisensory integration across different groups of healthy participants and clinical populations and in various task setting. This review will give an overview of the experimental evidence supporting the illusion, the current state of research concerning the influence of cognitive processes on the illusion, the neural mechanisms underlying the illusion, and future research directions. Moreover, an exemplary experimental setup will be described with different options to examine perception, alongside code to test and replicate the illusion online or in the laboratory.

## The Sound-Induced Flash Illusion

Multisensory integration is a fundamental perceptual process, by which information arriving from different senses is combined to a unified percept, and numerous studies showed that multisensory integration is beneficial for perception. For example, redundant multisensory information reduces reaction times (Miller, 1982; Cappe et al., 2009; Pomper et al., 2014). Moreover, the cocktail party effect, in which auditory perception is supported by visual cues, indicates that multiple streams of information can support decoding relevant information (Zion-Golumbic et al., 2013). However, multisensory information can also have detrimental effects, in which the perception of one sensory modality is affected by conflicting information from a second modality. Often, these incongruent information streams will be perceptually integrated, resulting in subjective illusions. Examples of such illusions due to incongruent multisensory information include the McGurk Effect (McGurk and MacDonald, 1976) or the Ventriloquist Effect (Choe et al., 1975). In both examples, visual information influences auditory perception. Interestingly, the unisensory information underlying these illusions is salient and easily perceived in isolation.

The above-mentioned examples illustrate an influence of visual information on auditory perception. However, the reverse influence has also been observed: Twenty years ago, Shams, Kamitani and Shimojo (Shams et al., 2000) published an – in their words – “striking visual illusion” indicating that visual perception can be influenced by other sensory modalities. The authors described that pairing a single visual stimulus with multiple auditory stimuli will lead to the illusory perception of multiple visual stimuli. This phenomenon was later coined the “sound-induced illusory flash effect” or “sound-induced flash illusion” (SIFI) (Bhattacharya et al., 2002; Shams et al., 2005a). The SIFI is a highly reliable effect that has been replicated in numerous studies. Interestingly, it is not specific to audiovisual stimuli, but the visual illusion can be induced by tactile stimuli as well (Violentyev et al., 2005). Yet, despite the overall robustness, there is a large inter-individual variability in the susceptibility to the illusion (de Haas et al., 2012). Across samples, the average likelihood of the illusion has often been reported to be around 50% for audiovisual (e.g., Keil et al., 2014) and visuotactile (e.g., Lange et al., 2013) stimuli. Thus, pairing one visual stimulus, which is easily detected in isolation and readily distinguished from two visual stimuli, with two auditory or tactile stimuli, renders visual perception bistable, but the individual likelihood to perceive the illusion varies.

A large number of empirical studies have explored the phenomenological change in perception, the underlying computational principles and the neural mechanisms associated with the perception of the illusion. After 20 years, it is time to summarize the current state of the research in a comprehensive review. Therefore, the aims of this review are to outline the proposed explanations of the SIFI, to compile current lines of research and to provide an update on future directions. Moreover, it will describe an example procedure to induce the SIFI alongside reproducible code for easy replication of the behavioral phenomenon.

## An Example SIFI Experiment

**FIGURE 1:**
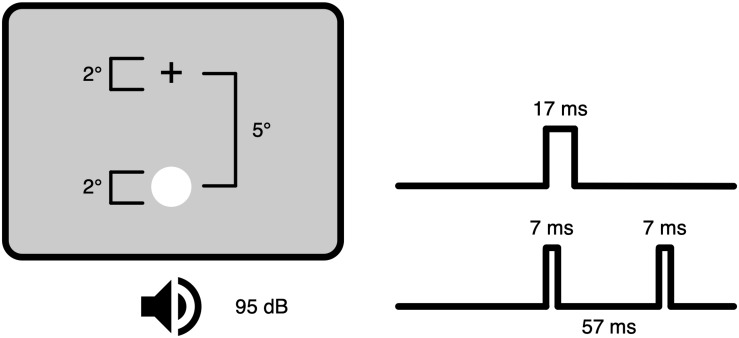
Experimental setup in the sound-induced flash illusion. A white flash is presented below a fixation cross outside of the foveal area on a neutral background. Simultaneously, two auditory beeps are presented. The duration of the visual stimulus is 17 ms, the auditory stimuli have a duration of 7 ms with an onset asynchrony of 57 ms.

The original publication on the SIFI (Shams et al., 2000) described an experiment comprising white disks subtending 2° at an eccentricity of 5° on black background spaced 50 ms in time and undefined beeps spaced 57 ms in time. Critically, observers reported multiple flashes, when one disk was accompanied by multiple beeps. A follow-up publication (Shams et al., 2002) further specified the luminance of the visual stimuli as 108 cd/m^2^ with a duration of 17 ms and the loudness of the auditory stimuli as 95 dB SPL at 3500 Hz with a duration of 7 ms (Figure 1). Thus, both the visual and auditory stimuli were highly salient. Interestingly, the authors claim that the specific stimulus characteristics should not influence the illusion perception, but the likelihood to perceive the illusion can be influenced by various stimulus and task characteristics (see section “Principles of Multisensory Integration and the SIFI”).

An examination of the optimal temporal spacing of the first and second auditory and visual stimuli indicated that this illusion declined above an offset of 70 ms. However, changing the offset between auditory and visual stimuli within ±70 ms did not affect the strength of the illusion (Shams et al., 2002). Therefore, multiple experiments used stimulus offsets of 180 ms to control for reaction tendencies not due to multisensory integration (Mishra et al., 2007; Michail and Keil, 2018). As a measure of perception, participants are usually asked to report the number of perceived flashes using a N-alternative forced-choice task, where N refers to the possible visual stimuli used in the experiment (e.g., 3-alternative forced-choice task: 0, 1, and 2). From this, the susceptibility to the illusion can be computed, as described in the next section (“Examining the Participants’ Perception”).

Based on this information, an example experiment was built using the open-source online experimental platform lab.js^1^. This experiment comprises 10 ms 1000 Hz tones in an individually chosen loudness in combination with visual stimuli subtending 1.6° at 4.1° eccentricity at a viewing distance of 60 cm with a duration of 16 ms (one screen refresh cycle at 60 Hz) on a neutral gray background. Importantly, due to the limitations of the lab.js platform, the onsets of the first auditory and visual stimuli are asynchronous, with the visual stimulus leading by one screen refresh cycle. The experiment can be tested at https://open-lab.online/test/sound-induced-flash-illusion-example/, and the code is available at https://github.com/juliankeil/SIFI_Example alongside an example analysis script for R.

In the last years, LED monitors have replaced CRT monitors in many laboratories. LED monitors in general provide less exact onset timing and stimulus duration (Elze, 2010; Cooper et al., 2013; Ghodrati et al., 2015). It is therefore important to consider the limits of the experimental setup and to design the experiment accordingly. Whereas it is recommended to use monitors and sound cards with precise timing in order to exactly control the onset and duration of the auditory and visual stimuli, empirical research shows that the SIFI is tolerant to asynchronies within ±70 ms (Shams et al., 2002). Extending psychophysical research to online studies will likely lead to less controlled stimulation environments due to the use of various combinations of hardware and software (Bridges et al., 2020). Therefore, presenting uni- and multisensory control conditions in laboratory and online studies is critically important, as these stimuli offer the possibility to check whether the participants can differentiate single and multiple auditory and visual stimuli and are actually reporting their perception.

### Examining the Participants’ Perception

The presentation of different combinations of flashes and beeps can either result in the so-called fission or fusion illusion (Andersen et al., 2004; Kaposvari et al., 2014). The fission illusion refers to the SIFI as described in the original publications by Shams et al. (2000, 2001, 2002). Here, two auditory stimuli are paired with one visual stimulus, which can result in the perception of two visual stimuli. The fusion illusion occurs following the presentation of one auditory stimulus together with two visual stimuli. Here, the illusion consists of the perception of only one visual stimulus.

Examining the participants’ subjective perception of the incongruent multisensory stimuli can be accomplished by asking the participants to rate their perception using a forced-choice task. From this rating, the individual susceptibility to the illusion can be computed. One often-used straightforward approach to this is the computation of the perception rate, i.e., the fraction of responses to a given stimulus combination indicating a certain perception relative to the number of presented stimuli (e.g., number of times the participant reported “2” relative to the number of presentations of one visual and two auditory stimuli). Alternatively, measures of perception based on the signal detection theory have been proposed (Watkins et al., 2006; Whittingham et al., 2014). Therein, information from different congruent and incongruent stimulus combinations is combined to compute sensitivity (d’) and response criteria. Vanes et al. (2016) describe how information from congruent trials with two beeps in combination with two flashes and incongruent trials with two beeps combined with one flash can be used in the analysis of the fission illusion. Importantly, participants report the number of perceived flashes and the authors consider “false alarms” as the illusion. Thus, the response of “2” in congruent trials (2 flashes-2 beeps) is a “hit,” and the response of “1” is a “miss.” Accordingly, the response of “2” in incongruent trials (1 flash-2 beeps) is a “false alarm” (i.e., fission illusion), and the response of “1” is a “correct rejection.” All these values are then considered relative to the number of presented trials in each condition. From these values, sensitivity can be computed as

$$d^{\prime }=z\,\left(\mathrm{hit}\,\mathrm{rate}\right)-z\,\left(\mathrm{false}\,\mathrm{alarm}\,\mathrm{rate}\right),$$

with z as the inverse of the standard normal cumulative distribution function. The criterion can be computed as,

$$\ln\left(\beta \right)=\left[z\,{\left(\mathrm{false}\,\mathrm{alarm}\,\mathrm{rate}\right)}^{2}-z\,{\left(\mathrm{hit}\,\mathrm{rate}\right)}^{2}\right]/2$$

Similarly, information from congruent trials with one beep in combination with one flash, and incongruent trials with one beep and two flashes can be used in the analysis of the fusion illusion. In this case, the response of “1” in congruent trials (1 flash-1 beep) is a “hit,” and the response of “2” is a “miss.” Accordingly, the response of “1” in incongruent trials (2 flashes-1 beep) is a “false alarm” (i.e., fusion illusion), and the response of “2” is a “correct rejection.”

In summary, presenting various combinations of one and two auditory and visual stimuli allows examining the fission and fusion illusion. The exact stimulus properties appear to be less critical. However, the presentation of the visual stimuli in the periphery and the temporal spacing of stimuli within ±70 ms are important. From the different stimulus categories, the response rate, the sensitivity, and the criterion can be computed as outcome parameters.

## Principles of Multisensory Integration and the SIFI

When we integrate multisensory information and perceive our environment, we have to solve two problems. On the one hand, we need to decide whether two signals come from a common source and integrate the signals accordingly. Over the course of a wide range of studies, three basic principles of multisensory integration have been established that guide our perception: the spatial principle, the temporal principle, and the principle of inverse effectiveness (Stein and Meredith, 1993; Stein et al., 2014). In short, these principles state that multisensory integration is strongest when the input modalities are spatially concordant, temporally aligned, and when the neural responses to the presented stimuli are weak. On the other hand, we need to estimate the reliability of the signals with respect to a given feature. In addition to the three principles of multisensory integration, the modality appropriateness hypothesis has been proposed (Welch and Warren, 1980). This hypothesis has been extended in a maximum-likelihood-estimation framework, which suggests that information from each sensory modality is weighted based on its relative reliability (Ernst and Bülthoff, 2004). Similarly, the information reliability hypothesis proposes the dominance of the modality providing the most reliable information (Andersen et al., 2004). With respect to the SIFI, the auditory system has a higher temporal resolution than the visual system. Because the auditory modality is more reliable, it should dominate the overall percept in the SIFI. Finally, Andersen et al. (2004) argue, that all these principles “should be considered as factors which contribute to the relative dominance of each modality and not as all-or-nothing principles.” At the same time, these authors highlight the role of cognitive processes such as attention in the SIFI and question the automatic multisensory integration. As a further extension of the maximum-likelihood-estimation framework, Shams et al. (2005b) compared the behavior of human participants to an ideal observer model and found that participants used Bayesian inference to decide whether, to what degree, and how to integrate the audiovisual information.

**FIGURE 2:**
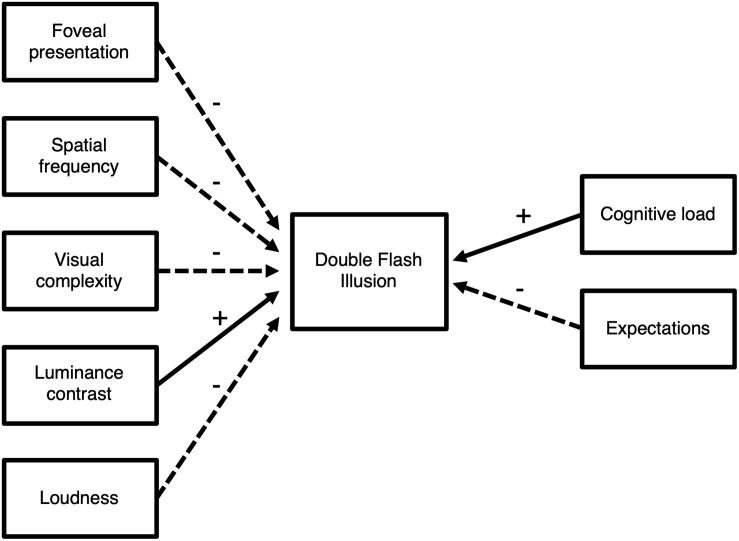
Overview of the influence of stimulus and task characteristics on the illusion rate. Experimental evidence indicates that various manipulations of the constituting stimuli and the task increase (+, solid arrows) or decrease (–, dashed arrows) the likelihood of an illusion.

With respect to the reliability of the input modalities, it should be noted that whereas presenting the visual stimulus in the fovea reduces the illusion rate (Shams et al., 2001), minor changes in size, eccentricity or luminance result in a similar perception of the illusion (e.g., Keil et al., 2014; Balz et al., 2016a; Michail and Keil, 2018). However, characteristics of the visual stimulus do appear to influence the illusion. For example, increased spatial frequency and visual complexity of the visual stimulus reduce the illusion perception, whereas increased luminance contrast has the opposite effect (Takeshima and Gyoba, 2013; Gyoba and Takeshima, 2015; Pérez-Bellido et al., 2015). Moreover, reducing the loudness of the auditory stimuli appears to reduce the illusion rate (Andersen et al., 2004; Figure 2).

In the SIFI, the constituting stimuli in the SIFI are presented in close temporal and spatial proximity. Moreover, compared to the auditory system, the temporal resolution of the visual system is relatively low (Vanrullen, 2016). In the SIFI, visual perception of short flashes is influenced by concurrent information from sensory modalities with a higher temporal resolution, such as auditory or tactile stimuli. Thus, multisensory integration in the SIFI follows the basic principles summarized above.

### Cognitive Influences on Multisensory Integration

The implicit assumption in studies on the principles of multisensory integration is that properties of the stimuli and the perceptual system guide conscious perception, and that this perception can be modeled using computational processes. However, Andersen et al. (2004) highlight the role of attention in the perception of the SIFI and recent behavioral studies underline that the perception of the SIFI can be influenced by cognitive factors.

Using the fusion and the fission illusion, Andersen et al. (2004) examined the influence of task instructions on behavior. They found that the integration of auditory and visual information was not automatic but depended on whether participants were instructed to count beeps or flashes. They thus interpreted their findings as support for the directed attention hypothesis, stating that the attended modality dominates perception. Using a task-independent modulation of cognitive resources, Michail and Keil (2018) examined the influence of cognitive load on the perception of the SIFI. The authors found that increased cognitive load induced by an n-back task leads to higher illusion rates. They argue that their findings provide strong evidence that audiovisual integration can be modulated by the amount of available cognitive resources and it therefore argues against a pre-attentive account of multisensory integration. A recent study examined the influence of expectations regarding the presented stimuli on the perception of the SIFI (Wang et al., 2019). In short, the authors show that expectations regarding the proportion of SIFI trials shape perception, indicating an influence of task-related cognitive processes.

Taken together, the available evidence supports the idea that multisensory integration in the SIFI is not an automatic and rigid process, but that the stimulus characteristics, task instructions and cognitive processes such as attention and expectations shape multisensory integration (Figure 2).

## Neural Mechanisms

The cognitive mechanisms summarized above suggest that the influence of auditory information on visual perception should occur at a later, decision-level stage rather than on an early sensory processing stage. Similarly, based on findings from the ventriloquist illusion, Rohe and Noppeney (2015) argue that unisensory stimuli are processed under the assumption of independence in early sensory processing stages, and that assumptions regarding the reliabilities of the signals are taken into account at higher processing stages. This idea is in line with findings from electroencephalography (EEG) experiments on the SIFI, which indicate that the perception of the SIFI is associated with increased gamma-band power. Gamma-band power has been interpreted as a signature of multisensory integration (Senkowski et al., 2005). Importantly, the increased gamma-band power related to the perception of the SIFI occurred relatively late, i.e., after the initial stimulus processing (Bhattacharya et al., 2002; Mishra et al., 2007; Balz et al., 2016a). Similarly, magnetencephalography (MEG) and EEG studies found increased evoked responses at longer latencies (Shams et al., 2001; Keil et al., 2014). Further support for a central role of higher-order cortical areas in the SIFI comes from studies using transcranial magnetic stimulation (TMS). Stimulating the angular gyrus resulted in a reduced likelihood to perceive the illusion and thus reduced multisensory integration (Kamke et al., 2012; Hamilton et al., 2013).

In contrast, some MEG and EEG studies also found earlier modulations of evoked responses and neural oscillations, indicating crossmodal influences on the level of early sensory cortical areas (Shams et al., 2005a; Mishra et al., 2007; Lange et al., 2011; Balz et al., 2016b). Similarly, an fMRI study found increased BOLD activity in the primary visual cortex (Watkins et al., 2006). However, the authors also note an involvement of the superior temporal sulcus and the inferior colliculi.

**FIGURE 3:**
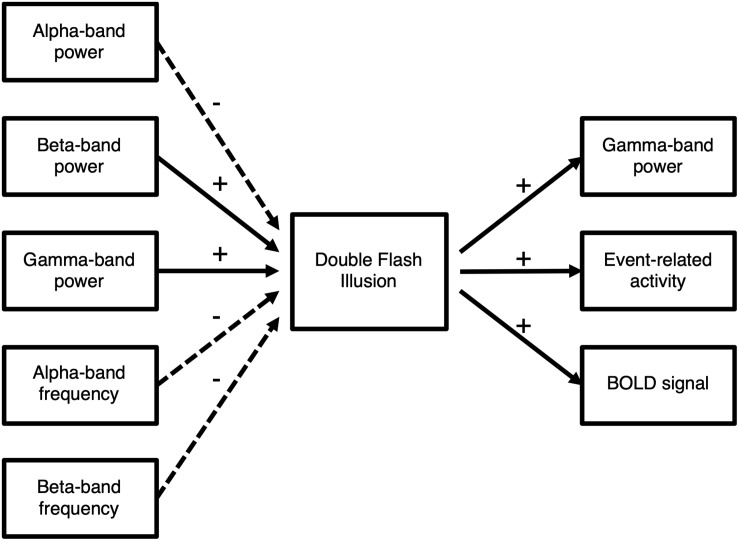
Overview of the neural mechanisms underlying the illusion. Experimental evidence indicates that the perception of the illusion is associated with increased gamma-band power, increased evoked responses, and increased bold activity (+, solid arrows). Moreover, higher beta- and gamma-band power increase the likelihood to perceive the illusion. In contrast, lower alpha-band band power and lower alpha- and beta-band frequencies increase the likelihood to perceive the illusion (–, dashed arrows).

Taken together, these findings on the neural substrate of the SIFI underline a multisensory integration process involving early and late processing stages at different hierarchies, in which crossmodal influences can influence perception at multiple stages (Senkowski et al., 2008; Keil and Senkowski, 2018; Figure 3).

## Neural Activity Influences Multisensory Perception

In everyday life, we constantly and effortlessly integrate the sensory inputs of our environment. In this regard, we are often able to predict future events. How we predict a future event has been discussed extensively at the theoretical level (Summerfield and Egner, 2009), and according to the predictive coding framework (Friston, 2005), we continuously uses available information to predict forthcoming events and to reduce sensory uncertainty. Importantly, this framework posits that top-down processes prior to stimulus presentation act upon primary sensory cortices. Recent studies have shown that sensory information is continuously sampled and that low-frequency oscillatory activity likely mediates this sampling (Busch and Vanrullen, 2010; Vanrullen et al., 2011). In addition, human studies have demonstrated that the amplitude and phase of oscillatory activity, as well as neural connectivity in cortical networks, relates to cognitive processes, sensory representation, attentional selection, and dynamic routing of information (Van Dijk et al., 2008; Mazaheri et al., 2009; Jensen and Mazaheri, 2010). These findings are in remarkable agreement with the results of animal studies (Fries, 2005; Schroeder and Lakatos, 2009).

Similar to unisensory stimulation, a number of studies have indicated that ongoing oscillations in cortical networks affect the processing of forthcoming multisensory stimuli. An MEG study examined the differences in oscillatory power and functional connectivity between SIFI trials in which the fission illusion was perceived and those trials in which only one visual stimulus was perceived (Keil et al., 2014). The authors report that increased beta-band power in the STG preceded a multisensory illusion, and that increased beta-band functional connectivity between STG and primary auditory cortex was related to illusion perception on a single trial level. Similarly, Kaiser et al. (2019) analyzed single-trial power prior to the SIFI and found that increased beta- and gamma-band power in occipital electrodes predicted the illusion perception. Moreover, using visual and tactile stimuli, Lange et al. (2013) found that reduced alpha-band power in visual cortical areas and increased gamma-band power in parietal and temporal cortical areas preceded the illusion, and the authors argued that this reflects cortical excitability. Two further studies highlighted the role of the alpha-band phase as a temporal window of integration (TWI) for the shaping of audiovisual perception (Cecere et al., 2015; Keil and Senkowski, 2017). The former authors found a correlation between the individual alpha-band frequency and illusion rate, which indicates that alpha-band oscillations provide a TWI in which the crossmodal influence could induce an illusion. Importantly, modulating the individual alpha-band frequency using transcranial alternating current stimulation modulated the probability of an illusion perception. Keil and Senkowski (2017) confirmed the relationship between the individual alpha-band frequency and the SIFI perception rate and localized this effect to the occipital cortex. Importantly, a recent EEG study on the auditory and tactile induced double flash illusion further confirmed the relationship between neural oscillations and the TWI (Cooke et al., 2019). However, whereas the authors replicated the relationship between the individual alpha-band frequency the SIFI, the TWI in the tactile-induced flash illusion was defined by the individual beta-band frequency. Thus, it appears that the neural oscillations recorded in visual cortex reflect task-depended functional connectivity networks. Interestingly, an MRI study found a correlation between the individual susceptibility of the illusion and the gray matter volume in the primary visual cortex (de Haas et al., 2012).

In agreement with studies on unisensory perception, a number of studies indicate that neural oscillations influence multisensory processing. Therein, alpha-band power indicates excitability in primary sensory areas and the phase of neural oscillations provides a TWI for crossmodal influence. Increased beta-band and gamma-band power in multisensory cortical areas might indicate increased readiness to integrate information (Figure 3).

## Clinical Applications and Future Directions

Empirical research over the last 20 years has established the SIFI as a robust and reliable tool to study multisensory integration in various settings and contexts. From this research, we can distill a standardized experimental setup and we have a mature sense of the average response to incongruent multisensory stimulation across various populations. Moreover, findings on the neuroscientific studies give us a detailed insight into the neural mechanisms underlying the illusion and their influence on perception. Based on this empirical background, we can now start to look into the factors influencing conscious perception. This includes brain states as well as cognitive factors and inter-individual differences in healthy and clinical populations.

In the last decade, empirical findings have indicated that the brain state influences information processing (Van Dijk et al., 2008; Busch and Vanrullen, 2010; Samaha et al., 2017). Importantly, multisensory studies have shown that cortical activity in one sensory area can influence information processing in other sensory cortical areas (Lakatos et al., 2007; Kayser et al., 2008; Mercier et al., 2015). Simultaneously, recent electrophysiological studies point toward a central role of neural oscillations and functional connectivity for multisensory integration and conscious perception (Lange et al., 2014; Keil and Senkowski, 2018). However, only few studies have examined causal manipulations of neural activity, for example using brain stimulation (Kamke et al., 2012; Hamilton et al., 2013; Cecere et al., 2015). Future studies on the SIFI could now be directed at changes in local cortical activity and network configuration associated with the illusion, and subsequent directed manipulation of these parameters.

Recent studies examined the influence of cognitive factors on multisensory integration. However, the role of attention therein is hotly debated (Andersen et al., 2004, 2009; Talsma et al., 2010; Macaluso et al., 2016; Tang et al., 2016), and the role of expectations for multisensory integration has only recently been examined (Gau and Noppeney, 2015; Wang et al., 2019). Behavioral data suggest that participants can adjust the susceptibility to the SIFI according to the expectations of stimulus timing (Chan et al., 2020). Moreover, concurrent emotional stimuli reduce multisensory integration, but information on the underlying neural mechanisms is scarce (Maiworm et al., 2012). Finally, the role of cognitive load for multisensory perception has only received little attention (Michail and Keil, 2018). Thus, future studies could aim to examine multisensory integration using a standardized experimental setup while manipulating cognitive influences. Concurrent examination of neural activity can then indicate the involvement of various cortical areas therein.

In addition, a number of studies have shown age-related changes in multisensory integration and perception (Murray et al., 2016; Hirst et al., 2018, 2020). Overall, these studies indicate that multisensory integration is plastic and dynamic across development from early life to adulthood. With respect to the SIFI, Hernández et al. (2019) found that the susceptibility to the illusion increased with age and declining cognitive status. Similar results indicate that cognitive impairment increases the susceptibility to the illusion across longer SOAs (Chan et al., 2015). It has been shown that cognitive performance varies as a function of the time of day (Monk et al., 1997; Carrier and Monk, 2000), but the influence of time on multisensory integration remains largely unexplored. Preliminary evidence indicates that neural oscillations, especially in the alpha band, vary with increased time on task (Benwell et al., 2019), yet the effects of these changes on multisensory integration have not been explored. Similar to the influence of cognitive processes on multisensory integration, using a standardized experimental setup in different age groups, at different times of the day, and at multiple replications can indicate developmental changes in multisensory integration and the influence of environmental factors therein.

In recent years, a number of studies have examined aberrant multisensory integration in various clinical populations. In autism spectrum disorder (ASD), deficits in perceiving the temporal relationship between different sensory inputs might impair multisensory integration. Accordingly, there is evidence of increased TWI in ASD, which is, however, more pronounced in speech stimuli than in the simpler SIFI (Stevenson et al., 2014). Similarly, along a psychosis continuum, increased proneness to the SIFI has been suggested to be linked to reduced temporal sensitivity. In a healthy population, Ferri et al. (2018) found increased susceptibility to the illusion in participants with high schizotypal scores, related to an increased TWI. Similarly, patients suffering from schizophrenia show an increased susceptibility to the illusion at longer SOAs, which is indicative of a lager TWI (Haß et al., 2016). However, no difference in the illusion perception appears at a short SOA, although changes in neural activity suggest aberrant multisensory processing in schizophrenia (Balz et al., 2016b).

Research on the SIFI uncovered basic neural and behavioral processes underlying multisensory integration. We now have a robust and reliable tool at our disposal to examine multisensory integration and perception in various settings. Future studies can now build upon these findings to further examine the influence of cognitive and emotional processes, development and aging, as well as fatigue and time on multisensory integration and perception.

## Author Contributions

JK conceived and wrote the manuscript.

## Conflict of Interest

The authors declare that the research was conducted in the absence of any commercial or financial relationships that could be construed as a potential conflict of interest.

## Footnotes

1 https://labjs.readthedocs.io/en/latest/
